# *In Vitro* Sensitivity of Paired *Leishmania* (*Viannia*) *braziliensis* Samples Isolated before Meglumine Antimoniate Treatment and after Treatment Failure or Reactivation of Cutaneous Leishmaniasis

**DOI:** 10.1155/2015/943236

**Published:** 2015-01-31

**Authors:** Cibele Baptista, Luciana de Freitas Campos Miranda, Maria de Fátima Madeira, Leonor Laura Pinto Leon, Fátima Conceição-Silva, Armando de Oliveira Schubach

**Affiliations:** ^1^Núcleo de Biossegurança, Bio-Manguinhos, Fundação Oswaldo Cruz, 21040-900 Rio de Janeiro, RJ, Brazil; ^2^Laboratório de Pesquisa Clínica e Vigilância em Leishmanioses, Instituto Nacional de Infectologia Evandro Chagas, Fundação Oswaldo Cruz, 21040-900 Rio de Janeiro, RJ, Brazil; ^3^Laboratório de Bioquímica de Tripanosomatídeos, Instituto Oswaldo Cruz, Fundação Oswaldo Cruz, 21040-360 Rio de Janeiro, RJ, Brazil; ^4^Laboratório de Imunoparasitologia, Instituto Oswaldo Cruz, Fundação Oswaldo Cruz, 21040-360 Rio de Janeiro, RJ, Brazil

## Abstract

This study evaluated the *in vitro* sensitivity of paired *Leishmania braziliensis* samples isolated from the same patient before pentavalent antimonial treatment (Sample A) and after treatment failure or cutaneous leishmaniasis reactivation (Sample B) in patients undergoing intralesional administration or injections (5 mgSb^V^/kg/d) of meglumine antimoniate. Fourteen samples from 7 patients were studied. After 24 h of drug exposure, 50% lethal dose (LD_50_) values for promastigotes ranged from 0.37 mg/mL to 5.86 mg/mL for samples obtained before treatment (A) and 0.89 mg/mL to 7.80 mg/mL for samples obtained after treatment (B). After 48 h, LD_50_ values ranged from 0.37 mg/mL to 5.75 mg/mL and 0.70 mg/mL to 7.68 mg/mL for A and B samples, respectively. After 48 h, LD_50_ values for amastigotes ranged from 11.7 to 44.3 *μ*g/mL for A samples and 13.7 to 52.7 *μ*g/mL for B samples. Of 7 patients, 1 discontinued treatment and 6 were cured after retreatment with amphotericin B (4 cases) or meglumine antimoniate (2 cases). Overall the B samples had higher LD_50_ values than A samples; however the difference was not significant. These results do not support the hypothesis that low-dose and intralesional treatments induce selection of resistant parasites *in vitro* and suggest that other factors may influence therapeutic outcome in patients with poor response to initial treatment.

## 1. Introduction

American cutaneous leishmaniasis (ACL) is caused by protozoan species of the genus* Leishmania* transmitted by the bite of infected phlebotomine sandflies [1]. In Brazil,* Leishmania (Viannia) braziliensis* is the main etiologic agent of cutaneous, mucosal, and mucocutaneous ACL [2–4].* Leishmania* sp. is a heteroxenous parasite with two developmental forms: amastigotes and promastigotes. Amastigotes are rounded and intracellular, and they are found in the parasitophorous vacuole of phagocytic mononuclear cells, especially macrophages, of vertebrate hosts. Promastigotes, in contrast, are elongated with free flagellum, and they develop in the gut of the sandfly insect vector as well as in axenic cultures. Both forms are used in* in vitro* assays to assess therapeutic sensitivity [5].

Pentavalent antimonials (Sb^V^) are the drugs of choice for treatment of cutaneous leishmaniasis (CL), with recommended doses of 10–20 mg Sb^V^/kg/d for 20 days [6]. For many years, the Evandro Chagas National Institute of Infectious Disease/Oswaldo Cruz Foundation (INI/FIOCRUZ) in Rio de Janeiro, Brazil, has administered 5 mg Sb^V^/kg/d intramuscularly continuously or in series [4, 7–9] or via intralesional (IL) administration [10, 11]. Treatment response is usually favorable in Rio de Janeiro regardless of regimen. Nevertheless, treatment failure or reactivation of skin lesions after treatment has been reported in various endemic areas [5, 6, 12–14]. Authors have associated treatment failure or reactivation with host immune response, pharmacological factors such as drug absorption and perfusion at the infection site, and, especially, factors associated with parasite resistance to antimonials [15, 16].

Antimonial resistance has been reported [16–19] and it should be considered a significant problem due to the limited drug arsenal for treatment of this disease [20].* Leishmania (Leishmania) donovani *isolates resistant to Sb^V^ have been identified in regions of India and Nepal. Resistance has been reported recently in the New World [21]. Efforts have been made to compare clinical treatment response to* in vitro* antimonial sensitivity. However, available* in vitro* techniques cannot detect* L. braziliensis* resistance with certainty [5, 22, 23].

In this study, we evaluated the* in vitro* susceptibility of* L. braziliensis* amastigotes and promastigotes to meglumine antimoniate by comparing paired isolates obtained from the same patient before and after treatment failure or reactivation of skin lesions.

## 2. Materials and Methods

### 2.1. Patients and Samples

Eligible patients included those undergoing CL treatment of 30 continuous intramuscular doses of 5 mg Sb^V^/kg/d meglumine antimoniate, 10-dose intramuscular series with 10 days rest between sets (low dose), or IL administration of the volume necessary to infiltrate the base of the lesion, with approximately 15 days between treatment applications. Two* Leishmania *(*V.*)* braziliensis* samples were isolated from the same lesion at diagnosis before treatment (Sample A) and after treatment failure or lesion reactivation (Sample B).

Treatment failure was defined as no progressive lesion healing after treatment completion. Reactivation was defined as lesion reactivation after apparently successful initial treatment with signs of healing.

All patients were from the state of Rio de Janeiro and attended the Leishmaniasis Outpatient Clinic at INI/FIOCRUZ.

The study was approved by the Ethics Committee in Research of the INI/FIOCRUZ. All patients signed a consent form prior to clinical evaluation to provide lesion samples for culture. Lesion fragments were seeded in Novy-MacNeal-Nicolle (NNN) biphasic medium and Schneider's* Drosophila* Medium (Sigma, St. Louis, Missouri) supplemented with 10% fetal calf serum (FCS) and antibiotics (200 U penicillin + 100 *μ*g streptomycin). Isolates were identified by isoenzyme electrophoresis and maintained in culture only through the fifth passage to maintain parasite infectivity.

### 2.2. Drug

Meglumine antimoniate (Glucantime, Sanofi-Aventis), Lot 604898, available in 5 mL ampoules containing 81 mg Sb^V^/mL, was provided by the Health Surveillance Department of the Ministry of Health, Brazil. The drug was diluted in Schneider's or RPMI-1640 (Gibco, BRL, Grand Island, NY, USA) culture medium for use in* in vitro* assays. Promastigotes and amastigotes from each sample were used to evaluate the* in vitro* drugsensitivity.

### 2.3. Promastigote Assays

First, sample growth curves were generated: test tubes (16 × 150 mm) containing 4 mL Schneider medium supplemented with 10% FCS and antibiotics were inoculated with 1 × 10^7^ parasites/mL and stored at 26–28°C. Quantification was performed in triplicate at 24 h intervals for 5 days using a Neubauer chamber and Trypan Blue staining.

Based on these growth curves, parasites in stationary phase and before their fifth passage in culture were used for sensitivity tests. Assays were performed in 96-well culture plates and evaluated after 24 and 48 h exposure to meglumine antimoniate. A and B samples were evaluated on the same plate and at the same time. A 100 *μ*L suspension containing 1 × 10^6^ parasites diluted in Schneider medium was placed in each well of a plate containing the same volume of drug (100 *μ*L). Twofold serial dilutions of meglumine antimoniate were used, starting at 8.1 mg Sb^V^/mL to 3.955 *μ*g Sb^V^/mL. The plates were incubated in a biological incubator (26–28°C), and the parasites were quantified after 24 and 48 h using a Neubauer chamber and Trypan Blue staining. A and B parasite samples not exposed to drug were used as controls. The experiment was performed in triplicate and values compared to no-drug controls. The dose of drug required for 50% parasite mortality (LD_50_) was determined from these measurements and calculated using Microsoft Excel software as described in Machado et al. [24].

### 2.4. Amastigote Assays

Amastigote sensitivity tests were conducted by* in vitro* infection of cultured murine macrophages. Briefly, the macrophages were isolated from peritoneal cavity of outbred Swiss Webster mice by washing with about 10 mL of RPMI-1640 medium using a syringe. These cells were plated (2 × 10^6^ macrophages/mL) in chamber slides (Lab-Tec, Nalge Nunc International) and then incubated for 2 h at 37°C in a 5% CO_2_ atmosphere. Nonadherent cells were removed by washing with RPMI-1640 medium, supplemented with 10% FCS. Cells were maintained under the same culture conditions for 24 h before infection. After this period, 5–10 promastigotes (Samples A and B) per macrophage were added. After 2 h, free parasites were removed by washing the monolayers with serum-free medium and the culture medium (RPMI + 10% FCS) was renewed. The drug at concentrations of 20, 40, and 80 *μ*g Sb^V^/mL, diluted in the same medium, was added 24 h after infection, with the infection kinetics evaluated at 24, 48, and 72 h. At each time point, the slides were washed with phosphate buffered solution (PBS) pH 7.2 (37°C), fixed with methyl alcohol, and stained with Giemsa. Controls were macrophages infected with Samples A and B without meglumine antimoniate. A total of 100 random macrophages at each time point from Samples A and B and their respective controls were counted under an optical microscope to determine the effect of drug concentration. The percentage (%) of infected cells and average number of amastigotes per cell were used to calculate infection rate. LD_50_, expressed at 48 h of infection kinetics, was calculated from a dose-response graph using GraphPad Prism (version 5.04).

### 2.5. Statistical Analysis

SPSS Statistics for Windows (version 17.0) was used to perform the Wilcoxon test to compare promastigotes and amastigotes from A and B samples; *P* < 0.05 were considered statistically significant.

## 3. Results

A total of 14 paired samples (A and B) from 7 patients were included in this study. Patient ages ranged from 18 to 71 years; 4 were women. Following initial treatment, treatment failure and reactivation were observed in 5 and 2 patients, respectively. Of these 7 patients, 1 patient discontinued treatment and 6 were cured after retreatment with amphotericin B (4 cases) or meglumine antimoniate (2 cases).

The promastigote growth curve revealed a stationary phase between days 3 and 4; parasites on the third day of growth were therefore used in the assays. Paired samples (A and B) from the same patient showed similar growth profiles. Except for 1 patient, A samples showed the highest mean number of parasites at all points of the curve compared to B samples. However, there were no differences in their murine macrophage infective capacity.

We observed a drastic reduction in the percentage of infected cells and the average number of intracellular amastigotes in most samples and all drug concentrations (20, 40, and 80 *μ*g/mL) at 72 h in the amastigote assays. For this reason, we used the 48 h time point to calculate LD_50_.

Table 1 shows patient data regarding treatment and outcome, as well as LD_50_ values for Sample A and B promastigotes and amastigotes forms. No significant difference was found between sample sensitivity levels.

## 4. Discussion

Pentavalent antimonials have been used to treat leishmaniasis with variable efficacy for about 70 years; resistance has been reported, particularly in visceral leishmaniasis [17, 18, 20]. In this study, we evaluated 14 paired* L. braziliensis* samples to verify the association between* in vitro* susceptibility and treatment outcome in patients treated with meglumine antimoniate.

Hypotheses on the development of parasite antimonial resistance gained prominence from publications by Grogl and colleagues [16, 25] based on results of* in vitro* assays that suggested that inadequate therapeutic doses could induce selection of antimonial-resistant parasite clones. This observation was strengthened by accounts of Sundar et al. [26] in India, where there was a failure to control the use of antimonials for treatment of visceral leishmaniasis. Lira et al. [27] showed that* L. donovani* isolated from patients in India that were nonresponsive to antimonials had 3-fold higher LD_50_ values than isolates from drug-responsive patients. In another study using paired samples, samples isolated after treatment showed the higher LD_50_ values compared to samples taken before treatment [28]. Despite speculation, there is still no definitive marker for parasite antimonial resistance [29].

The patients in our study were diagnosed, treated, and followed up at INI/FIOCRUZ, which has a long and successful treatment history using intramuscular injections (5 mg Sb^V^/kg/d) or IL administration of meglumine antimoniate [4, 8–11]. Of 7 patients enrolled in this study, 5 were treated with intramuscular administration of the low-dose regimen and 2 received IL treatment. Retreatments were administered at the discretion of the treating physician. Regarding therapeutic outcomes, 1 patient stopped treatment. Two patients were cured with the same treatment regimen initially employed (5 mg Sb^V^/kg/d), and 4 patients were cured with retreatment using amphotericin B [6].

Because other factors may be involved, it is often difficult to associate therapeutic failure only to parasite resistance [22, 23, 30]. However, knowledge of characteristics of parasites isolated in different situations can contribute important elements to this discussion.

Parasite species and subpopulations with genetic polymorphisms may also influence clinical course and treatment response.* L. braziliensis* is known toconsist of populations with high genetic variability that can cause predominantly cutaneous and mucosal lesions. However, although different clinical patterns and varied treatment response are reported in the state of Rio de Janeiro, the* L. braziliensis* genetic profile is homogeneous [31]. The samples in this study also had homogeneous phenotypic profiles, without isozyme variation.

Growth curves were generated for all samples to determine timing of the stationary phase. This phase was reached between the third and fourth days of growth for all samples, as also reported by Moreira et al. [23]. Paired samples (A and B) from the same patient showed similar growth profiles. Growth profile differences could also be due to sample heterogeneity; further consideration of* in vitro *growth parameters is necessary [32]. An interesting observation in our study was that, except for 1 patient, A samples showed the largest average number of parasites at all points of the curve compared to B samples. This finding suggests that prior exposure to treatment could impair the ability of promastigotes to multiply* in vitro*. However, A and B samples showed no difference in their ability to infect murine macrophages.

The variable* in vitro* susceptibility of promastigotes and amastigotes may be related to experimental conditions or inherent characteristics of evolutionary forms. A significant limitation of using promastigotes in these tests is that they are not the evolutionary form in the vertebrate host. Similar to our findings, others have reported promastigotes to be resistant to meglumine antimoniate, requiring higher drug doses than amastigotes [5]. Vermeersch et al. [33] propose that an intracellular amastigote model should be the standard reference for* in vitro* sensitivity testing. Although our results revealed large heterogeneity in LD_50_ values, they generally agree with other studies using promastigotes [5, 32]. It was not possible to establish a relationship between therapeutic response and* in vitro *sensitivity data with promastigotes because 2 of 4 patients with increased Sample B LD_50_ recovered after meglumine antimoniate retreatment. This suggests that other variables may have positive or negative influences in this context. Zauli-Nascimento et al. [21] also found no correlation between* in vitro* results and therapeutic response in patients with ACL.

In addition to the large variation of LD_50_ absolute values in both promastigotes and amastigotes, we found that B samples had higher LD_50_ values compared to A samples. This result might suggest that samples isolated after reactivation are less sensitive to meglumine antimoniate* in vitro*; however, the difference was not statistically significant. These results do not support the hypothesis that low dose or IL treatments induce selection of resistant parasites* in vitro*. Other factors such as immune response to infection may influence treatment outcome in patients with poor response to initial treatment; correlations should therefore be treated with caution.

Zauli-Nascimento et al. [21] also found no correlation between* in vitro* results and therapeutic response in patients with ACL. According to these authors, there is no evidence of primary parasite resistance to Sb^V^ in Brazil, unlike reports in other endemic areas. Because treatment response to ACL is multifactorial, different approaches should be considered and additional studies using samples from responder and non responder patients should be encouraged. Further studies using larger numbers of isolates and new markers could add to the results of this study and contribute to a better understanding of the mechanisms involved in Sb^V^ resistance.

## Figures and Tables

**Table 1:** Clinical outcome related to treatment of tegumentary leishmaniasis, in which paired *Leishmania braziliensis* samples were analyzed by *in vitro* sensitivity testing. LD_50_ values for amastigotes (*μ*g Sb^V^/mL) and promastigotes (mg Sb^V^/mL) forms obtained before treatment (A) and after treatment failure or reactivation (B) are shown.

Patient code	Time until diagnosis^*^	Lesion number	1st treatment/clinical outcome	Interval between isolation of samples A and B^*^	Retreatments/clinical outcome	Amastigotes LD_50_ (*μ*g Sb^V^/mL) 48 h^**^		Promastigotes LD_50_ (mg Sb^V^/mL) 24 h^***^		Promastigotes LD_50_ (mg Sb^V^/mL) 48 h^****^	
						A	B	A	B	A	B
1	18	1	5 mg Sb^V^ series—30 doses/TF	21	2nd T: 5 mg Sb^V^ series 3rd T: IL 4th T: Amp B Healing	17.97	17.94	3.06	6.98	1.08	5.82

2	3	1	IL—2 doses/reactivation	13	2nd T: IL 3rd T: Amp B Healing	11.7	13.7	4.38	0.89	5.75	1.37

3	1.5	5	5 mg Sb^V^ series—30 doses/TF	14	2nd T: 5 mg Sb^V^ continuous 3rd T: 5 mg Sb^V^ continuous Healing	11.7	14.1	0.37	1.78	0.37	0.70

4	2	2	5 mg Sb^V^ continuous—30 doses/TF	07	2nd T: 5 mg Sb^V^ continuous 3rd T: 15 mg Sb^V^ + pentoxifylline Treatment dropout	14.0	15.2	5.20	6.79	3.04	5.35

5	1.5	1	5 mg Sb^V^ continuous—38 doses/TF	18	2nd T: IL 3rd T: 5 mg Sb^V^ continuous 4th T: 5 mg Sb^V^ continuous 5th T: Amp B healing	44.3	52.7	5.86	7.80	3.28	7.68

6	Unknown	1	5 mg Sb^V^ continuous—45 doses/reactivation	27	2nd T: 5 mg Sb^V^ series 3rd T: 5 mg continuous Healing	16.1	33.7	4.53	7.46	3.00	5.61

7	1.5	1	IL—8 doses/TF	13	2nd T: Amp B Healing	16.8	16.3	5.05	7.70	1.04	3.56

^*^Time in months; T (treatment); IL (intralesional administration); Amp B (amphotericin B); TF (treatment failure); ^**^ *P* value = 0.063; ^***^ *P* value = 0.176; ^****^ *P* value = 0.128.
